# Advancements in Sample Preparation Methods for the Chromatographic and Mass Spectrometric Determination of Zearalenone and Its Metabolites in Food: An Overview

**DOI:** 10.3390/foods12193558

**Published:** 2023-09-25

**Authors:** Yifeng Lou, Qingyang Xu, Jiaqi Chen, Sen Yang, Zheng Zhu, Di Chen

**Affiliations:** 1 School of Pharmaceutical Sciences, Zhengzhou University, Zhengzhou 450001, China; lyf202153030615@stu.zzu.edu.cn (Y.L.); 15038130627@163.com (Q.X.); chenjiaqi@stu.zzu.edu.cn (J.C.); senyang@zzu.edu.cn (S.Y.); 2 Key Laboratory of Targeting Therapy and Diagnosis for Critical Diseases of Henan Province, Zhengzhou 450001, China; 3 National Key Laboratory of Cotton Bio-Breeding and Integrated Utilization, Zhengzhou University, Zhengzhou 450001, China

**Keywords:** zearalenone, sample preparation, extraction, chromatography, mass spectrometry

## Abstract

Zearalenone and its metabolites are mycotoxins generated by Fusarium species while crops are growing and can typically be found in various foods, posing a risk to human health. Governments have implemented stricter regulations concerning the permissible levels of zearalenone in food products to safeguard public health. Stricter regulations on zearalenone levels in food have been implemented. However, detecting zearalenone and its metabolites remains challenging due to sample complexity and interference. Surprisingly few reviews of sample preparation methods for zearalenone in food have appeared in the past decade. In this overview, we outline the most recent developments in the sample pre-treatment technology of zearalenone and its metabolites in food samples based on chromatography–mass spectrometry methods since 2012. This review covers some prominent technologies, such as liquid–liquid extraction-based methods, solid-phase extraction-based methods, and QuEChERS (quick, easy, cheap, effective, rugged, and safe) extraction, providing valuable insights into their advantages and limitations for potential applications. The assessment of the methods discussed, along with an overview of current challenges and prospects, will guide researchers in advancing the field and ensuring safer food quality for consumers worldwide.

## 1. Introduction

Zearalenone (ZEN) is a mycotoxin originating from specific Fusarium fungi, frequently detected in cereals and agricultural commodities like wheat, maize, barley, oats, and sorghum [1]. During the metabolism of ZEN in animals or food processing, it forms important metabolites, such as zearalanone (ZAN), α-zearalanol (α-ZAL), β-zearalanol (β-ZAL), α-zearalenol (α-ZEL), and β-zearalenol (β-ZEL), all of which have estrogenic activity [2]. These metabolites can specifically bind to estrogen receptors in domestic animals, thereby inducing a series of reproductive toxicities and teratogenic effects [2]. The impact of these phenomena encompasses the peroxidation of polyunsaturated fatty acids within the cellular membrane. Consequently, lipid peroxides emerge, causing subsequent disturbances in cellular metabolism and functionality, potentially culminating in cellular demise [1]. ZEN can also enter the human body through contaminated meat, milk, vegetable oil, and food products, causing obvious necrosis of peripheral blood mononuclear cells as well as adverse effects on the motility and function of sperm. These effects can impact the development of human reproductive organs and reproductive function [3]. As a result, the excessive intake of ZEN-contaminated food can negatively impact the well-being of both humans and animals [4].

**Figure 1:**
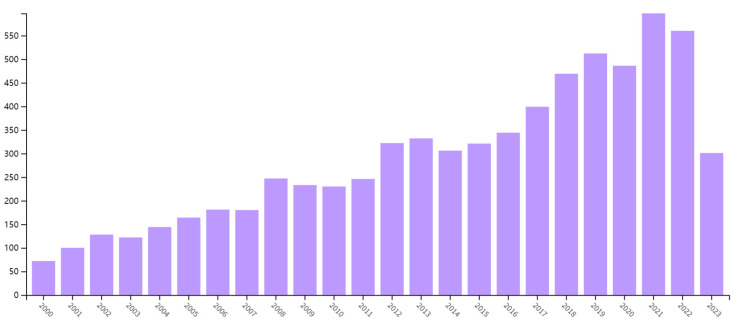
Change in the number of publications from 2000 for the topic category “zearalenone”.

The significant harm caused by zearalenone in corn has led to a surge in related research. A Web of Science search of scientific databases demonstrates the proliferation of zearalenone in academic literature since the year 2000, as depicted in Figure 1. The quantity of published papers incorporating zearalenone as a component of their research has increased over the years, with an annual average of more than 400 articles.

ZEN and its metabolites have been identified in 46 different foods, including corn, wheat, soybeans, beer, milk, cooking oil, and feedstuffs [5,6,7,8], prompting numerous countries worldwide to implement regulations on zearalenone levels [9]. For instance, China’s “Hygienical standard for feeds” (GB 13078-2017) sets a maximum allowable zearalenone content of 500 ng/g in corn feed, while the “Limits of Mycotoxins in Food” (GB2761-2017) prescribes that the content of zearalenone in wheat shall not exceed 60 ng/g [10,11]. Similarly, the European Commission specifies that all feedstuffs must have zearalenone limited to 5–20 ng/g. At the same time, the allowable limit of zearalenone in corn and grain is 100 ng/g, with a tolerable daily intake of 0.25 μg/kg body weight [12,13]. Given the potential health hazards associated with zearalenone and its metabolites and the existing regulations, strict monitoring and control of these compounds in food and feed are imperative to ensure food safety and animal wellbeing [14].

To effectively detect ZEN and its metabolites in food samples, various analytical techniques have been developed, including chromatography, mass spectrometry, immunoassay, Raman spectroscopy, electrochemical methods, and spectrophotometry [15,16,17,18,19,20,21,22]. However, these analytical techniques present certain limitations. For instance, distinguishing metabolites in immunoassays may be challenging due to potential false-positive effects caused by immunoaffinity reagents [23]. Raman spectroscopy may yield weak signals, making it difficult to distinguish certain samples [24]. Electrochemical analysis may suffer from poor selectivity [25], while spectrophotometry’s accuracy is relatively low [26]. In contrast, mass spectrometry and chromatography have become the most widely used detection methods, offering high efficiency, speed, resolution, micro-detection, and automation performance [27]. They can simultaneously detect multiple components with excellent selectivity and sensitivity. A variety of chromatographic methods, including capillary electrophoresis, liquid chromatography (LC), and gas chromatography (GC), combined with various detectors, have been employed to determine zearalenone and its metabolites [28,29,30]. Additionally, mass spectrometry, either alone or in combination with chromatographic separation, is suitable for the analysis of zearalenone and its metabolites [18].

Mass spectrometry and chromatography are crucial tools in mycotoxin detection due to their precision, sensitivity, and reliability [31]. While chromatographic and mass spectrometry techniques offer numerous advantages, directly applying them to analyze zearalenone and its metabolites in food samples proves challenging [32,33]. The complexity and diversity of chemical compounds in food matrices, coupled with the low analyte concentrations, pose practical difficulties in determining zearalenone and its metabolites without adequate sample preparation. Matrix interference further hampers successful detection and quantification of zearalenone at low abundance levels [34]. Moreover, incompatibilities may arise between complex food samples and instrumental analysis. To address these challenges, researchers have turned to effective sample preparation methods as a crucial step prior to instrumental detection. The use of effective sample preparation methods not only enhances the sensitivity of zearalenone and its metabolite detection but also helps in improving the overall accuracy and reliability of the analysis. By concentrating the analyte and eliminating interfering substances, these methods ensure more precise quantification and reliable results. As a result, these advancements in sample preparation techniques have led to significant progress in zearalenone and its metabolite analysis in food samples, ultimately contributing to the assurance of safer food quality for consumers worldwide.

**Figure 2:**
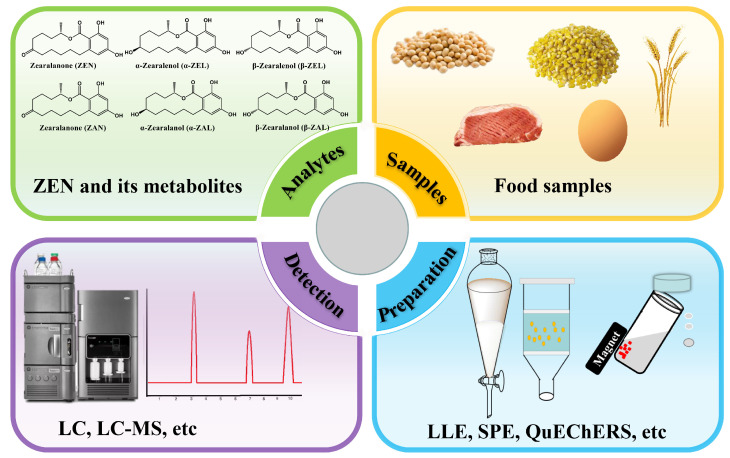
A diagram outlining a standard analytical procedure for identifying ZEN and its metabolites in samples of food.

At present, various methods for preparing samples have been developed, often in combination with mass spectrometry and chromatography techniques, to facilitate effective sample analysis. These methods include solid-phase extraction (SPE), QuEChERS, liquid–liquid extraction (LLE), and related methodologies derived from them [34,35,36,37,38]. Research continues to explore and optimize these methods, paving the way for further advancements in mycotoxin toxin testing and food safety analysis. Each sample preparation technology has its own applications, merits, and limitations. Proper sample preparation plays a crucial role in increasing the likelihood of successful sample analysis. This paper reviews the most recent advancements in sample preparation techniques for the analysis of ZEN and its metabolites in food samples, covering the period from 2012 to the present (see Figure 2). Table 1 lists the abbreviations and definitions used throughout this review.

## 2. Sample Preparation Methods

### 2.1. Liquid–Liquid Extraction-Based Methods

LLE is widely employed in the preparation of samples for food analysis. It relies on the principle that the analyte exhibits different partition coefficients between two immiscible solution bodies or phases [37]. LLE involves the use of an organic solvent that does not mix with water. After the extraction liquid is dried, the residue can be dissolved in a more suitable solvent for analysis and determination. It is currently the most widely used separation and purification technology, offering advantages such as a high recovery rate, large processing capacity, continuous operation, good separation efficiency, and easy automatic control. However, traditional LLE also has disadvantages, including time-consuming procedures, complex operations, and the consumption of large amounts of organic solvents. Additionally, it is less effective with highly polar analytes. To address these limitations, microextraction techniques like liquid–liquid microextraction (LLME) and dispersive liquid–liquid microextraction (DLLME) have been widely adopted. These techniques offer several advantages, including improved cost-effectiveness, high recovery, and efficient enrichment [35,36]. Analytical techniques such as GC, LC, and others, in combination with LLE, LLME, or DLLME techniques, have been used to determine ZEN and its metabolites in food commodities (Table 2).

DLLME comprises two steps: first, it involves extracting and dispersing the analyte; then, it requires centrifugation of the resulting mixture. The extraction takes place within a ternary blend consisting of an extraction solvent, a dispersion solvent, and an aqueous sample [49]. The nature of the dispersion solvent is crucial, as it disperses the fine drops of the extractant in the water medium. The choice of the appropriate extraction solvent is a key factor in the success of the extraction process. It affects both extraction recovery and selectivity. As an illustration, Pi et al. [40] utilized this technique by using 600 μL of 1-dodecanol as the extractant and 1.0 mL of acetonitrile (ACN) as the dispersant. They employed a method that combined ultrasonic-assisted aqueous two-phase extraction with solidifying organic drop-dispersive liquid–liquid microextraction. This approach enabled the simultaneous determination of nine mycotoxins, including ZEN, in medicinal and edible foods using LC with an in-series diode array detector (DAD) and fluorescence detector (FLD) [40].

In recent years, there has been a growing recognition of the importance of developing analytical methods that align with the principles of green chemistry in the field of food analysis. Green chemistry aims to minimize the environmental impact of chemical processes and promote sustainability. In the context of sample preparation methods for the chromatographic and mass spectrometric determination of zearalenone and its metabolites in food, it is crucial to consider the environmental implications of the techniques employed. This includes the choice of solvents, reagents, and extraction procedures. One notable approach that contributes to greener sample preparation is the use of deep eutectic solvents (DES). These solvents are formed by mixing two or more components, typically a hydrogen bond acceptor and a hydrogen bond donor, at a specific ratio that results in an eutectic mixture with a significantly lower melting point than the individual components. DES offer several advantages that make them attractive alternatives to traditional organic solvents. They possess low volatility, non-flammability, and biodegradability, thus reducing the release of harmful substances into the environment. Additionally, DES can be easily prepared from readily available and renewable components, further enhancing its sustainability.

**Figure 3:**
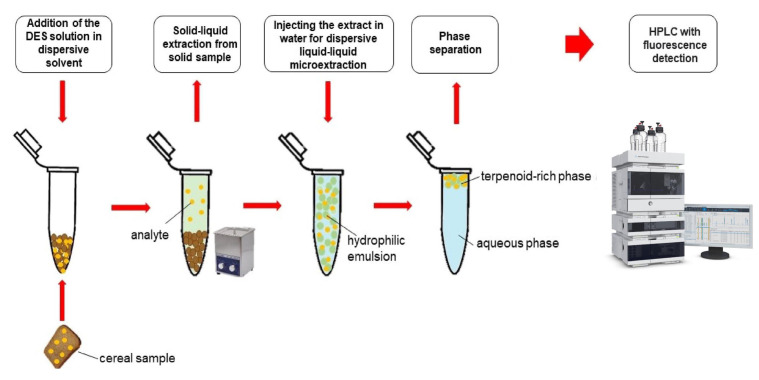
Ultrasound-assisted solid–liquid extraction in combination with DLLME using deep eutectic solvents. Figure modified from [39].

By incorporating DES as an extraction solvent in the chromatographic and mass spectrometric determination of zearalenone and its metabolites in food, researchers can significantly reduce the environmental impact of the analytical process. DES not only provides improved extraction efficiency and selectivity but also minimizes the generation of hazardous waste. These characteristics make DES a promising option for achieving greener sample preparation and aligning with the principles of green chemistry. Pochivalov et al. [39] successfully used the analytical method of dispersive liquid–liquid microextraction gas chromatography flame ionization detector (DLLME-GC-FLD) to determine the content of zearalenone in grain samples. They used terpenoids and long-chain alcohols as extraction solvents and ACN as a dispersion solvent to separate dispersed solvents with smaller organic phase volumes (as shown in Figure 3). The DES extraction with DL-menthol and 1-hexanol as raw materials had a good effect, with a recovery rate of 93 ± 4% and an enrichment coefficient of 15.8 ± 0.7. The sensitivity of this method was also impressive, with a limit of detection (LOD) as low as 2 ng/g, highlighting the effectiveness of DES as a green and efficient extraction solvent for analytes in various samples. This makes it a promising choice for environmentally friendly and sensitive analytical applications.

To achieve higher extraction efficiency, it is essential to select suitable micro-extraction conditions based on the analyte. These factors include the volume and type of dispersion solvent, extraction solvent, and extraction time [50], as well as the viscosity, surface tension, and dielectric constant of the solvent. Generally, the dispersive solvent should dissolve in the extraction solvent but be insoluble in water, while the extraction solvent should be immiscible with water. It should have a low boiling point, a lower density than water, and be easily separable. Since choosing appropriate dispersion and extraction solvents is crucial for improving extraction efficiency, various solvents are often tested to optimize extraction conditions when developing extraction technologies. The specific extraction conditions may vary for different sample substrates. For instance, Emidio et al. [36] determined zearalenone in water samples using the DLLME-LC–MS/MS (tandem mass spectrometry) method. The optimal extraction conditions were as follows: 100 μL of bromocyclohexane was used as the extraction solvent (non-dispersible solvent), 10 mL of water sample (adjusted to pH 4), vortex extraction for 2 min, and centrifugation at 3500 rpm for 10 min without adjusting ionic strength. The LODs and limit of quantifications (LOQs) were in ranges of 4–20 ng/L and 8–40 ng/L, respectively. The results demonstrated that the method was suitable for determining ZEN in water samples [36].

Due to the fact that centrifugation can prolong sample preparation time, there is increasing interest in developing non-centrifugation methods to accelerate the extraction process. One such innovative approach gaining attention is solidifying organic drop-dispersive liquid–liquid microextraction (SOD-DLLME) without the need for centrifugal steps [51,52]. A novel method based on SOD-DLLME was reported for the simultaneous determination of nine mycotoxins in edible and medicinal foods using LC with DAD and FLD in series. The optimized conditions for the method involved using 600 μL of 1-dodecanol as the extractant, 1.0 mL of ACN as the dispersant, and a vortex-assisted time of 1.0 min. LODs for DAD and FLD were determined to be 0.1622 ng/mL and 0.04451 ng/mL, respectively.

DLLME is usually used to prepare liquid samples and is not usually used to prepare solid samples. However, the limitations of DLLME for solid samples can be overcome by combining it with other sample preparation methods such as SPE and QuEChERS [38,44]. For example, Zhou et al. [45] prepared samples by combining SPE with DLLME, used chloroform as the extraction solvent, and then conducted LC-FLD analysis to determine zearalenone in grains and beans. In addition, auxiliary energy fields such as ultrasonic and eddy currents can be added to the DLLME to replace artificial vibration with mechanical vibration to improve the efficiency of the dispersion step.

In recent years, ionic liquids have become increasingly popular as extraction solvents in DLLME because of their good properties, including high density, high thermal stability, low volatility, low water solubility, and toxicity. Bozkurt et al. [6] proposed a DLLME method utilizing ionic liquids and used this technology to extract zearalenone from beer and grain samples. Methanol was used as the dispersion solvent, while 1-butyl-3-methylimidazolium bis(trifluoromethanesulfonyl)imide and 1-methyl-3-octylimidazolium bis(trifluoromethanesulfonyl)imide were utilized as the extraction solvents [6]. By coupling with LC-FLD, the LOD of zearalenone was 0.25 ng/mL. In addition, Wang et al. [41] developed a method for zearalenone analysis in corn products by combining vortex-assisted ionic liquid dispersion liquid–liquid microextraction with LC-FLD. In the experiment, 10 g of a fully homogeneous corn sample and 50 mL of a methanol/water (80:20, *v*/*v*) mixture were extracted in the ultrasonic cleaning machine for 30 min, and the extraction solution was filtered using filter paper as a dispersion and [HMIM][PF6] as an extraction solvent. The LODs and LOQs were 0.3 and 1.0 ng/g, respectively. The average recoveries ranged from 83.5% to 94.9%, and the relative standard deviation (RSD) was less than 5.0%, showing that this method was applicable to the detection of zearalenone in corn products [41].

Recently, Ni et al. [42] measured zearalenone in corn oil by combining the immunomagnetic beads technique with the DLLME technique. Immunomagnetic beads have many advantages, such as easy surface modification, uniform particle size, and large specific surface area. Meanwhile, the purification method of immunomagnetic beads has the advantages of strong specificity, simple operation, and fast separation speed. By combining the advantages of immunomagnetic beads and DLLME technology and using LC-FLD for quantitative detection and analysis, zearalenone in concentrated corn oil was directly purified under the solubilization of the surfactant. With the characteristics of automation and high efficiency, the pre-treatment method of this technology is more environmentally friendly [42].

While LLE-based methods are recognized for their ease of use and efficiency, they can lead to environmental pollution due to the organic solvents used. Additionally, the potential for cross-contamination arises when dealing with samples of complex composition. To address these concerns, advancements in this method should prioritize environmentally friendly and sustainable practices, such as exploring greener alternatives like DES, which can enhance analyte selectivity while maintaining environmental integrity. By adopting such strategies, researchers can contribute to the development of more eco-friendly and sustainable sample preparation methods for the chromatographic and mass spectrometric determination of zearalenone and its metabolites in food.

### 2.2. Solid-Phase Extraction-Based Methods

**Figure 4:**
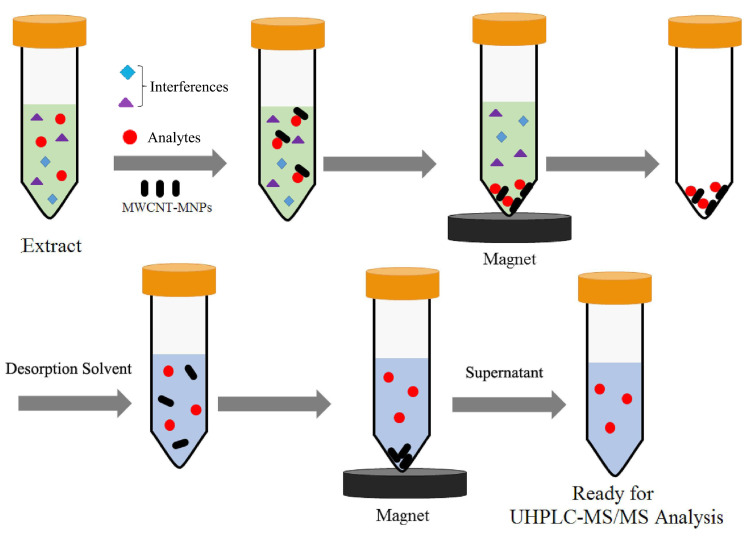
Schematic diagram of the magnetic solid-phase extraction (MSPE) procedure based on multi-walled carbon nanotubes-magnetic nanoparticles (MWCNT-MNPs). Figure modified from [55].

SPE employs a solid adsorbent to capture the desired compound within the liquid sample. This process effectively isolates the target compound from any interfering substances present in the sample or its matrix. Subsequently, the compound is eluted using an eluent or through thermal desorption. This elution process serves the purpose of separating and concentrating the target compound (see Figure 4). SPE has undergone significant development in many related branches, such as dispersive solid-phase extraction (d-SPE), solid-phase microextraction (SPME), magnetic solid-phase extraction (MSPE), and stir-bar sorptive extraction (SBSE); all are receiving increasing attention [53,54]. SPE is a superior alternative to LLE due to its enhanced selectivity, reduced solvent usage, automation-friendly nature, and minimal cross-contamination risk. It enables efficient pre-concentration, sample cleanup, and compatibility with various detection techniques, making it a preferred choice in modern analytical chemistry.

Although these pre-treatment technologies have greatly improved the sample yield in terms of solvent consumption, extraction time, enrichment factor, and reproducibility, conventional adsorbents (such as octadecyl silicon and polymer reverse phase materials) in common use lack good selectivity [56]. This is an analytical deficiency that requires extensive optimization to minimize matrix adsorption/interference and co-elution of non-target compounds. Obviously, it is very important to develop a better selective adsorbent for the detection of zearalenone and its metabolites. In the chemical structure of zearalenone, the seventh carbon contains a separate ketone group, which is easy to react with a hydrazine group to form zearalenone hydrazone on the solid phase. Drzymala et al. [57] combined dynamic covalent hydrazine chemistry with LC-FLD analysis technology to quantitatively detect the content of zearalenone in edible oil. The resin skeleton, particle size, pore size, specific surface area, resin skeleton, and load performance of seven different hydrazine materials were evaluated. Therefore, we chose a hydrazine-based functional silica gel. Decoupling was achieved by introducing 20% acetone, which reacted with the hydrazine group, thereby replacing and releasing zearalenone for detection. The final experimental results indicated that the LOD is 10 ng/g and the LOQ is 30 ng/g. It is worth noting that by applying a mixture of acetic acid (AcOH) and methanol, the recovery and activation of the hydrazine cylinder can be achieved in one step. Therefore, acetone was removed, and the hydrazine portion was converted into the corresponding acetate. A hydrazine cartridge can be repeatedly applied 15 times without affecting its performance [57].

In recent years, nanomaterials have played an increasingly important role in the fields of chromatography and mass spectrometry technology. To some extent, magnetic nanoparticles are very helpful for extraction and preconcentration techniques, mainly because they can be easily separated from the matrix through external magnets without retaining the remaining magnetization. Due to their exceptional physical and chemical properties (among other good properties) of iron oxides (magnetite, Fe_3_O_4,_ and maghemite, γ-Fe_2_O_3_), they have become the most widely used magnetic nanoparticle in d-SPE as well as in other applications. Gonzalez-Salamo et al. [58] synthesized Fe_3_O_4_@pDA m-NPs in their laboratory. Prior to liquid chromatography–mass spectrometry (LC–MS) analysis, it was used as a d-SPE adsorbent to extract zearalenone and its metabolites from complex substrates such as milk (full-fat skimmed milk and semi-skimmed goat milk) and yogurt (an unsweetened natural yogurt). Because this kind of food sample matrix is complex, it is not possible to directly apply previously developed methods. To reduce the matrix effect of the sample and the problem of contamination, clogging or damaging the LC column, etc., the team added the initial deproteinization step to remove the milk protein. The experimental results showed that when the sample was 1.5 mL of milk, the best removal effect of milk protein was obtained by using 3 mL of ACN and 75 μL of acetic acid, and a slightly higher reproducible relative recovery can be obtained by using 80 mg of Fe_3_O_4_@pDA NPs as adsorbent and 8 mL of methanol as eluent. For the yogurt sample, using the same volume of AcOH, change the sample/ACN ratio from 1/2 to 1/3 *w/v*. Under these conditions, taking 1.5 g of yogurt, 4.5 mL of ACN, and 75 μL of AcOH, a good deproteinization effect can be obtained. The amount of adsorbent and eluent extracted is the same as that of milk samples. Finally, they performed matrix matching calibration and recovery studies on the selected matrix. The results showed that the linearity was good, the relative recovery was in the range of 70–120%, the RSD was lower than 16%, the LOD of the milk sample was in the range of 0.21–4.77 ng/mL, and the LOD of the yogurt sample was in the range of 0.29–4.54 ng/g [58]. In another study, Zhao et al. [59] prepared PEGylated multi-walled carbon nanotube magnetic nanoparticles (PEG-MWCNTs-MNP) as adsorbents and used the MSPE method for sample pre-treatment. It was shown that when the sample was 4 g of liquid milk and 10 mg of adsorbent was used, the extraction efficiency was the highest. However, when the desorption agent was ethyl acetate containing 1% formic acid, the desorption efficiency was the best. The extracted zearalenone and its metabolites were quantified with LC-Q-Exactive HRMS. The results showed that the LOD and LOQ of liquid milk were in the range of 0.005–0.050 ng/g and 0.015–0.150 ng/g, respectively [59].

In addition to magnetic nanomaterials as adsorbents, many examples of different adsorbents used in these methods have been reported in the literature, including reduced graphene oxide and gold nanoparticle composites [60], graphitized carbon black [61], chitosan nanofibers [62], immunosorbents [63], and molecularly imprinted polymers (MIPs) [64,65]. Utilizing immunoaffinity columns (IACs) for mycotoxin purification and concentration has garnered significant research attention. The notable benefit of IAC lies in the exceptional specificity exhibited by imprinted antibodies towards their intended analytes. Regrettably, the majority of commercially accessible IAC contain antibodies tailored for just a single mycotoxin or a narrow cluster of closely associated mycotoxins, rendering them expensive and non-reusable. More in-depth studies are needed on the application of IAC to sample pre-treatment of zearalenone and its metabolites [66]. MIPs, being a synthetic polymer material, have demonstrated their excellence as adsorbents in the realm of molecularly imprinted solid-phase extraction (MISPE). This method offers considerable benefits, including specific molecular recognition, exceptional predetermined selectivity, remarkable stability, reusability, and the capacity to effectively mitigate background interference during detection. Moya-Cavas et al. [67] polymerized N-(2-aminoethyl) methylacrylamide as a functional monomer, methylacrylamide as a comonomer, ethylene glycol dimethacrylate as a crosslinking agent, and silicon beads as a sacrificial scaffold. The polymerized compound cyclododecyl 2,4-dihydroxybenzoate (CDHB) was similar to zearalenone in size, function, and shape and was used as a template substitute for MIP synthesis. The eluent was eluted with 2.5 mL of trifluoroacetic acid/methanol (3/97, *v*/*v*), and zearalenone was analyzed by LC-FLD. The results show that the LOD of the oil sample is 5 ng/g [67]. Zhang et al. [68] prepared hydroxyapatite-supported surface imprinted polymers (HAP@MIPs) using coumarin-3-carboxylic acid and naringenin as pseudotemplate molecules for zearalenone. They characterized the sorbent using various methods and found that it achieved adsorption equilibrium within 6 min, with an adsorption capacity of 6.77 µg/mg. HAP@MIPs were employed as the sorbent, and 3 mL of methanol was used as the eluent in SPE. This was coupled with LC to identify zearalenone in different cereal samples. The sample recoveries obtained ranged from 70.09% to 101.88%, with standard deviations of 2.06% to 8.47% [68]. Table 3 presents a variety of materials used as SPE sorbents for determining ZEN and its metabolites in food samples in recent years.

Ever since its inception as a technique for preparing samples, especially liquid samples, SPE-based approaches have found widespread application in separating, enriching, and purifying various compounds. This encompasses eliminating interfering substances [78]. The rapid progress of SPE-based methods can be attributed to the synthesis of numerous new sorbents in recent years. SPME requires fewer consumables, involves simpler handling, and encounters less interference from the sample matrix. Consequently, SPME can be a suitable alternative to SPE in many scenarios [18]. Future research in this field should prioritize the development of sorbents with enhanced selectivity and higher adsorption capacity while also aiming to reduce the amount of sorbent required for extraction. This reduction would contribute to minimizing or eliminating the reliance on organic solvents. Additionally, ensuring the repeatability and reproducibility of the obtained sorbents remains an important consideration.

### 2.3. QuEChERS

QuEChERS (quick, easy, cheap, effective, rugged, and safe), a swift sample preparation technique, emerged in recent years as an innovative advancement renowned for its speed, accuracy, and efficiency [79]. Since the introduction of the QuEChERS method, it has gained substantial popularity. It is widely employed for the qualitative and quantitative detection of pesticide residues in diverse agricultural products. Through extensive research, the QuEChERS method has found widespread application in various food testing scenarios [80].

**Figure 5:**
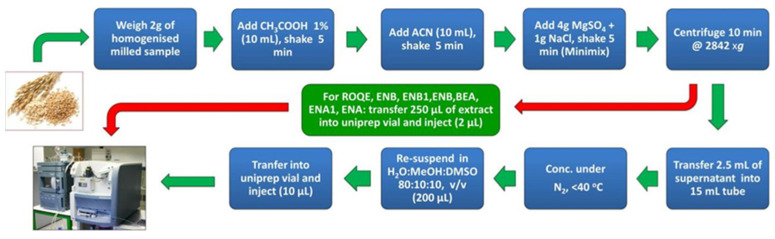
General procedure for analyzing zearalenone in cereals based on QuEChERS [82].

The typical procedure for the QuEChERS method is as follows: (1) sample grinding; (2) using ACN extraction separation; (3) adding MgSO_4_ and other salts for water removal; (4) adding adsorbents such as ethylenediamine-n-propyl silane to remove impurities; (5) detection of the supernatant by appropriate detection technique. The general procedure for this method is depicted in Figure 5. Compared to traditional sample preparation methods, QuEChERS offers several advantages, including fast analysis speed, minimal contamination, low cost, and high recovery. Originally designed for identifying pesticide residues in fruits and vegetables, the QuEChERS method has more recently found application in the analysis of zearalenone and its metabolites in food. This is frequently conducted in combination with LC and LC–MS/MS. He et al. [81] used an enzyme-linked immunosorbent assay (ELISA) and LC to detect ZEN after QuEChERS extraction. The optimal composition for QuEChERS extraction is 6 g of MgSO_4_, 1.5 g of sodium chloride, 1.5 g of sodium citrate dihydrate, and 1 g of citric acid sesquihydrate. The optimal adsorbent is 300 mg of primary secondary amine [81].

Ferreira et al. [82] developed a rapid sample preparation method that relied on the pre-extraction of fatty compounds using n-hexane, ACN extraction of target analytes, simple d-SPE using MgSO_4_ and C18 adsorbents to remove matrix co-extracts, followed by glycosylation and further gas chromatography–mass spectrometry (GC–MS) analysis of the analytes [83]. Prior to residue analysis, the authors verified the analytical method for LOD, LOQ, linearity, and recovery, all of which fell within acceptable ranges [82]. Table 4 demonstrates the application of QuEChERS to the extraction of ZEN and its metabolites in various food samples. These examples illustrate the suitability of QuEChERS for sample preparation in various analytical methods.

QuEChERS technology has gained widespread adoption across various fields since its inception. Its advantages of being fast, simple, inexpensive, efficient, reliable, and safe are widely recognized. However, there are still some challenges that need to be addressed. One such challenge is the limited sampling volume, which can impact the detection limit to some extent. Additionally, the use of ACN or acidified ACN for extraction in this technology can potentially cause instrument damage when combined with chromatographic techniques. Furthermore, the inherent limitations of the technology in eliminating matrix effects in complex matrices require additional correction processes, which may introduce errors in the analysis. Therefore, further advancements and improvements should be pursued in future studies. These may include exploring new purification agents, optimizing extraction reagents, and developing correction methods to mitigate the influence of matrix effects.

## 3. Selecting a Sample Preparation Strategy

As previously discussed, a variety of sample preparation methods have played a crucial role in preparing samples for the precise quantification and identification of zearalenone and its metabolites in diverse food samples, particularly those susceptible to mycotoxin contamination. Each of these methods comes with its own distinct advantages and disadvantages, necessitating careful consideration based on the specific circumstances and characteristics of the sample matrix. For instance, SPE is well-regarded for its exceptional selectivity and cleanliness, making it suitable for highly precise analyses, though it can be time-consuming and involve higher costs. On the other hand, the QuEChERS method offers a rapid and cost-effective solution, particularly ideal for screening purposes in extensive sample surveys, even though it may exhibit reduced selectivity and robustness. LLE, commonly chosen for its simplicity and efficiency, may have environmental concerns due to solvent use, but the employment of environmentally friendly solvents such as DES could address this issue. It is important to note that there is no one-size-fits-all method; suitability is paramount and depends on factors such as sample type, analysis cost, the specific goals of the research, and the technological resources available.

To determine the most suitable sample preparation method for a specific scenario, one must carefully weigh these advantages and disadvantages against the specific analysis requirements. Factors like sample complexity, analyte concentration, available resources, and the desired level of sensitivity and selectivity all play a pivotal role in method selection. Considering the potential presence of other mycotoxins or contaminants within the sample matrix is also crucial. By conducting a comprehensive assessment of these variables and tailoring the choice of sample preparation method accordingly, researchers can ensure the most accurate and effective determination of zearalenone and its metabolites in food, contributing to safer and more dependable food quality control measures.

## 4. Chromatographic and Mass Spectrometric Analysis

The complexity of food matrices will potentially interfere with the selectivity of zearalenone detection. Thus, apart from implementing effective sample preprocessing techniques for purification and enrichment prior to instrumental analysis, the choice of an appropriate detection instrument is equally essential. Presently, LC-FLD and LC–MS are extensively utilized for detecting ZEN in food samples [89,90]. However, LC-FLD detection relies on chromatographic separation of the analyte and coexisting matrix, which results in poor selectivity. In contrast, LC–MS offers high sensitivity and selectivity for analysis. LC–MS showcases exceptional sensitivity and unparalleled selectivity for analysis [91]. This inherent capability of LC–MS to precisely differentiate target compounds from background interferences increases its efficacy in ZEA and metabolite determination, thereby significantly increasing the reliability of results obtained. LC-FLD has lower analysis costs and can be used for preliminary screening. However, for more accurate results, it is usually necessary to confirm the potentially positive samples identified in the preliminary screening using LC–MS.

For both LC-FLD and LC–MS, optimal chromatographic separation conditions that distinguish the target analyte from the matrix are pivotal for enhancing analysis selectivity. When opting for an LC column to detect ZEN and its metabolites, the C18 column is the usual choice. Moreover, the utilization of an ultra-high-performance liquid chromatography (UPLC) column, featuring a smaller stationary phase particle size, has gained traction. This selection heightens column efficiency and yields satisfactory analytical outcomes, with higher resolution and narrower chromatographic peaks achievable in a shorter timeframe. The choice of the mobile phase significantly influences peak shape and separation effects. Typically, ACN and methanol in conjunction with water are widely employed for the reverse-phase chromatographic separation of ZEN and its metabolites. During LC–MS analysis, ZEN and its metabolites are commonly detected using negative ion mode, employing either ESI or APCI ionization methods. However, the complex nature of food matrices can greatly suppress the analyte ionization response, impacting the accuracy, precision, and uncertainty of detection in LC–MS analysis. Overcoming matrix effects is crucial for achieving accurate and reliable quantitative analysis in LC–MS [92].

For LC–MS analysis of food samples, using isotopically labeled internal standards for matrix effect correction is an ideal approach. Isotopically labeled analytes closely resemble the analytes of interest in structure and properties. Stable isotope dilution methods involve using molecules labeled with stable isotopes such as ^13^C, ^15^N, or ^2^H. These labeled molecules possess the same structure as the target analyte, serving as internal standards or diluents. This effectively minimizes matrix effects on the signal, offering high sensitivity in the nanogram or lower range. This method proves highly advantageous for the accurate quantitative analysis of zearalenone in food. The stable isotope dilution method has been successfully applied to detect zearalenone in various food products, including beer, maize, wheat flour, dairy products, peanut butter, food-grade gums, baby food, and animal feed, yielding positive results. For instance, Lijalem et al. [9] developed a stable isotope dilution-LC–MS to determine zearalenone and its derivatives in maize. They employed ^13^C or ^2^H -labeled molecules with the same chemical structure as the target mycotoxin as internal standards, coupled with LC–MS for detection [9]. This approach had good selectivity, sensitivity (LODs: 0.14 to 0.33 ng/g), accuracy (relative recoveries: 96.7–103.6%), and precision (RSDs < 4%) within the concentration range of 20–400 ng/g. Thus, meeting the requirements of most applications.

However, applying stable isotope dilution methods for zearalenone determination in food is hindered by the high costs associated with synthesizing stable isotope internal standards specific to zearalenone and its metabolites. Additionally, most of its metabolites lack commercially available isotope-labeled analyte standards. Therefore, recent advancements in stable isotope labeling strategies have aimed to address these limitations [93]. Zhang et al. [94] developed an isotope-coded derivatization method to quantify six mycotoxins, including ZEN, in various complex matrices using LC–MS/MS. This involved derivatizing both actual samples and standards with d_0_-dansyl chloride and its heavy counterpart, d_4_-dansyl chloride. A mixture of derivatives labeled with light and heavy isotopes was then analyzed using LC–MS/MS, allowing for isotopic internal calibration. This method achieved high sensitivity, with detection limits ranging from 31.03 to 38.15 ng/L. Accuracy percentages ranged from 97.13% to 104.1%, with precision below 3.42%. Successful application of this method was demonstrated by analyzing mycotoxins in three cereal samples (corn, wheat, and sorghum), yielding recoveries ranging from 71.3% to 92.6%. Future advancements in stable isotope labeling technology should focus on improving existing labeling techniques and reagents. Progress and refinement in isotope labeling technology are expected to greatly contribute to mycotoxin detection in food and enhance global food safety.

In addition to LC-FLD and LC–MS, GC–MS has also been employed for the detection of ZEN and its metabolites. However, in contrast, GC–MS necessitates the process of derivatization when analyzing polar ZEN and its metabolites [95]. This is due to the fact that ZEN compounds are nonvolatile and cannot be directly detected upon entering a gas chromatograph–mass spectrometer. Volatile derivatives are formed to enable their analysis, which is achieved by reducing the polarity of the target compounds through silylation derivatization. Principal silylation reagents, such as N,O-bis(trimethylsilyl)trifluoroacetamide (BSTFA), N-(trimethylsilyl)imidazole (TSIM), and N-methyl-N-trimethylsilyl trifluoroacetamide (MSTFA), are commonly employed for the chemical derivatization of ZEN compounds. As a result, this can result in GC–MS being relatively time-consuming. It might be precisely this reason that there are fewer studies utilizing GC–MS for the detection of ZEN and its metabolites compared to articles employing LC-FLD and LC–MS analytical techniques.

## 5. Conclusions and Future Perspectives

The precise determination of ZEN and its metabolites within food matrices has evolved into a critical imperative for upholding food safety standards. However, this task is far from straightforward due to the inherent challenges posed by factors such as the minute concentrations of ZEN and its metabolites in complex food matrices, exacerbated by the presence of an assay of interfering compounds. As a result, the necessity to enhance sample purity and enrichment for quantification emerges as paramount, ensuring the acquisition of dependable quantitative and qualitative insights. To tackle these complexities, a diverse array of sample preparation methodologies has found application in the analysis of zearalenone and its metabolites. Among these techniques, LLE and LLME, SPE and SPME, QuEChERS approaches, and derivatization strategies have gained widespread use. The recent integration of novel materials within microextraction methodologies has introduced streamlined, cost-efficient, and high-efficiency avenues for the analysis of zearalenone in food matrices. However, the horizon of zearalenone determination methods extends beyond current achievements, as researchers are propelling the field toward the amalgamation of distinct sample preparation techniques with automated, reproducible, and precision-oriented assays. An important facet of this trajectory lies in the challenge of miniaturizing the sample processing workflow. This must be achieved while preserving accuracy and robustness and reducing time expenditure, solvent usage, and costs. This formidable goal is underscored not only by economic considerations but also by a conscientious commitment to environmentally sustainable practices. In this evolving landscape, future advancements are poised to revolutionize zearalenone and its metabolite analysis, fostering enhanced food safety regulation and safeguarding public health against mycotoxin contamination.

## Figures and Tables

**Table 1:** Abbreviations used in this review article.

Abbreviation	Definition	Abbreviation	Definition	Abbreviation	Definition
ACN	acetonitrile	IMB_S_-DLLME	Dispersive liquid–liquid microextraction based on immunomagnetic beads	PEG-MWCNTs-MNP	PEGylated multi-walled carbon nanotubes magnetic nanoparticles
AcOH	acetic acid	IR-SPE-DLLLME	interference removal solid-phase extraction dispersive liquid–liquid microextraction	QuEChERS	Quick, Easy, Cheap, Effective, Rugged, and Safe
CDHB	cyclododecyl 2,4-dihydroxybenzoate	LC	liquid chromatography	RSD	relative standard deviation
CHCl_3_	chloroform	LC–MS/MS	liquid chromatography–tandem mass spectrometry	SBSE	stir-bar sorptive extraction
DAD	diode array detector	LLE	liquid–liquid extraction	SOD-DLLME	solidifying organic drop-dispersive liquid–liquid microextraction
DES	deep eutectic solvent	LOD	limit of detection	SPE	solid-phase extraction
DLLME	dispersive liquid–liquid microextraction	LOQ	limit of quantification	SPME	solid-phase microextraction
d-SPE	dispersed solid-phase extraction	MEKC-MS	micellar electrokinetic chromatography–mass spectrometry	UPLC	ultra-high-performance liquid chromatography
ELISA	enzyme-linked immunosorbent assay	MeOH	methanol	VA-IL-DLLME	vortex-assisted ionic liquid dispersive liquid–liquid microextraction
FID	flame ionization detector	MIA	multiple-impurity adsorption	ZAN	zearalanone
FLD	fluorescence detector	MIP	molecularly imprinted polymer	ZEN	zearalenone
GC	gas chromatography	MISPE	molecularly imprinted solid-phase extraction	α-ZAL	α-zearalanol
GC–MS	gas chromatography–mass spectrometry	MNPs-MWCNTs	magnetic nanoparticles coated with a layer of octadecyl group-modified silica containing multiwalled carbon nanotubes	α-ZEL	α-zearalenol
HAP@MIPs	hydroxyapatite-supported surface imprinted polymers	MS/MS	tandem mass spectrometry	β-ZAL	β-zearalanol
IAC	immunoaffinity columns	MSPE	magnetic solid-phase extraction	β-ZEL	β-zearalenol

**Table 2:** Application of liquid–liquid extraction-based (LLE) methods for the determination of ZEN and its metabolites in foods.

Sample	Sample Usage	Sample Preparation	Extraction Solvent	Analyte	Detection	Linear Range	Recovery	LOD	LOQ	Ref
Cereal	0.5 g	DLLME	1.5 mL DES (DL-menthol:1-hexanol = 2:1, mol/mol)	ZEN	GC-FID	5–500 ng/g	93 ± 4%	2 ng/g	5 ng/g	[39]
Medicinal and edible Food	1.0 g	SOD-DLLME	600 μL 1-dodecanol	ZEN	LC-DAD/FLD	0.5–200.0 ng/mL	82.31–104.3%	0.01563–0.5161 ng/mL	0.05210–1.720 ng/mL	[40]
Maize Products	10.0 g	VA-IL-DLLME *	100 μL [HMIM][PF_6_]	ZEN	LC-FLD	1.0–1000.0 ng/mL	83.5–94.9%	1.0 ng/g	0.3 ng/g	[41]
Corn oil	0.25 mL	IMBS-DLLME *	0.45 mL 2.5% PBST solution	ZEN	LC-FLD	10–1000 ng/g	85.7–89.0%	3.3 ng/g	10 ng/g	[42]
Rice bran	20 g	DLLME	295 μL chloroform	ZEN	LC–MS/MS	2.5–7.5 ng/g	46–107%	2.5 ng/g	5.0 ng/g	[43]
Corn	/	DLLME-μ-SPE	320 μL 1-heptanol	ZEN	Spectrofluorimetry	0.51–300 ng/mL	93.2–102.1%	0.25 ng/mL	/	[44]
Cereals, legumes, multigrain crops, etc.	2.5 g	IR-SPE-DLLLME *	498 μL chloroform	ZEN	LC-FLD	10–300 ng/g	63.22–93.21%	0.03–11 ng/g	0.10–44 ng/g	[45]
Beer	/	DLLME	75 μL chloroform	ZEN	LC-FLD	5–2000 ng/mL	71–108%	0.12 ng/mL	0.40 ng/mL	[46]
Amaranth seeds	2.0 g	DLLME-SFO-SBE	100 μL 1-dodecanol	ZEN	LC–MS/MS	0.65–500.00 ng/g	100.05%	0.21 ng/g	0.65 ng/g	[47]
Natural yogurt	2–4 mL	DLLME	110 μL chloroform	α-zearalenol	MEKC-MS	275–2500 ng/mL	/	5–29 ng/mL	/	[48]
Milk	2–4 mL	DLLME	110 μL chloroform	α-zearalenol	MEKC-MS	150–2500 ng/mL	/	1–61 ng/mL	/	[48]

* VA-IL-DLLME: vortex-assisted ionic liquid dispersive liquid–liquid microextraction; IMB_S_-DLLME: dispersive liquid–liquid microextraction based on immunomagnetic beads; IR-SPE-DLLLME: interference removal solid-phase extraction dispersive liquid–liquid microextraction.

**Table 3:** Application of solid-phase extraction-based (SPE) methods for the determination of ZEN and its metabolites in food.

Sample	Sample Volume	Analyte	Sorbent	Sample Preparation	Detection	Linear Range	Recovery	LOD	LOQ	Ref.
Milk samples	1.5 mL	ZEN, β-ZAL α-ZAL, β-ZEL α-ZEL, ZAN	Fe_3_O_4_@pDAm-NPs	MSPE	LC–MS	20–400 ng/mL	77–120%	0.21–4.77 ng/g	0.71–15.91 ng/g	[58]
Liquid milk	4.0 g	ZEN, α-ZOL, β-ZOL, ZAN, α-ZAL, β-ZAL	PEG-MWCNTs-MNP	MSPE	LC-HRMS	0.15–100 ng/mL	81.8–106.4%	0.005–0.05 ng/g	0.015–0.15 ng/g	[59]
Oil samples	2.0 g	ZEN	CDHB	MSPE	LC-FLD	100–500 ng/g	89%	5 ng/g	16 ng/g	[67]
Vegetable oil	2.0 g	ZEN	Magnetic nanoparticles coated with double layers of silicon dioxide	MSPE	LC–MS/MS	0.178–625 ng/g	89.4–93.0%	1.03 ng/g	/	[69]
Maize	6.0 g	ZEN, α-ZOL, β-ZOL, α-ZAL, β-ZAL, ZAN	MNPs-MWCNTs *	MSPE	LC–MS	4–40 ng/mL	91.6–98.3%	0.6–1.0 ng/mL	1.9–3.3 ng/mL	[70]
Corn	2.0 g	ZEN	Magnetic nanographene oxide	MSPE	LC-FLD	0.13–1.25 μg/mL	79.3–80.6%	0.05 μg/mL	0.13 μg/mL	[71]
Maize, milk, egg	2.0 g	ZEN, α-ZEL, α-ZAL, β-ZEL, β-ZAL	Fe_3_O_4_@TAPB-Tp *	MSPE	LC–MS/MS	0.1–100 ng/g	81.27–90.26%	0.003–0.018 ng/g	0.012–0.050 ng/g	[72]
Vegetable oil	1.0 g	ZEN	MIPs	SPE	LC-FLD	10–2000 ng/g	89–93%	1.10 ng/g	1 ng/g	[73]
Maize	5.0 g	ZEN	BONDESIL-SI, Cleanert IC-H, and Esela^®^ HLB	MIA*	LC–MS/MS	\	77.5–98.4%	0.3 ng/g	0.08 ng/g	[74]
Corn, Coix seed, Gualoupi	1.0 g	ZEN	Natural cotton fiber	In-syringe SPE	LC–MS/MS	0.1–100 ng/g	93.8–110.4%	0.083	0.025 ng/g	[75]
Corn juice	30 mL	ZEN, ZAN	Amphiphilic polymers	SPE	LC–MS/MS	0.05–20.00 ng/mL	85–93%	0.015–0.017 ng/mL	0.050–0.057 ng/mL	[76]
Milk	2 g	ZEN, α-ZAL, β-ZAL, α-ZEL, and β-ZEL	Hydrophilic covalent organic frameworks	SPME	Ambient mass spectrometry	0.1–100 ng/mL	80.58–109.98%	0.05–0.1 ng/mL	0.2–0.3 ng/mL	[18]
Corn, millet, coix lachryma	10 mL	ZEN	Fe_3_O_4_-HAP@MIPs	MSPE	LC-FLD	10–300 ng/g	61.97–95.15%	2 ng/g	6.65 ng/g	[77]

* MIA: multiple-impurity adsorption; MNPs-MWCNTs: magnetic nanoparticles coated with a layer of octadecyl group-modified silica containing multiwalled carbon nanotubes.

**Table 4:** Utilizing QuEChERS-derived techniques to analyze ZEN and its metabolites in diverse food samples.

Sample	Sample Usage	Analytes	Sample Preparation	Detection	Linear Range	Relative Recovery	LOD	LOQ	Ref.
Maize, wheat, rice	4.0 g	ZEN	QuEChERS	ELISA	13.64–104.48 ng/mL	83.55–102.49%	2.58 ng/L	/	[81]
Popcorn	5.0 g	ZEN	QuEChERS	GC–MS	10–200 ng/g	65–68%	7–16 ng/g	20–48 ng/g	[83]
Vegetable oils	1.0 g	ZEN, α-ZEL	QuEChERS	LC–MS/MS	2–100 ng/mL	78.2–104.6%	0.10–0.32 ng/g	0.37–1.05 ng/g	[84]
Bean products, flour products, milk	4.0 g	ZEN	QuEChERS	LC–MS/MS	\	77.9–118.0%	0.5–1.0 ng/g	/	[85]
Pig tissues (heart, liver, spleen, and muscle)	1.0 g	ZEN, ZAN, α-ZEL, β-ZEL, α-ZAL and β-ZAL	QuEChERS	LC–MS/MS	2–1000 ng/mL	70–110%	0.5–1 ng/g	1–2 ng/g	[86]
Milk	5.0 mL	ZEN, α-ZEL, β-ZEL, α-ZAL, β-ZAL	QuEChERS	LC–MS/MS	10–200 ng/mL	68–114%	0.5 ng/mL	1 ng/mL	[87]
Brown rice	1.0 g	ZEN	QuEChERS	LC–MS-MS	/	81–101%	0.85 ng/mL	2.6 ng/mL	[88]

## Data Availability

The data used to support the findings of this study can be made available by the corresponding author upon request.
